# Spatio-temporal differences and associations between upper and lower respiratory microbiota in ventilator-associated pneumonia

**DOI:** 10.3389/fcimb.2026.1757535

**Published:** 2026-06-05

**Authors:** Hong Yang, Xin-Xin Ni, Feng Xu, Xin-Yi Lu, Yu Chen, Jia-Le Chen

**Affiliations:** 1 Wuxi Maternal and Child Health Hospital, Wuxi School of Medicine, Jiangnan University, Jiangsu, China; 2 Department of Anesthesiology, Wuxi Huishan People’s Hospital, Jiangsu, China; 3 Hospital Infection Management Section, Wujin Affiliated Hospital of Nanjing University of Traditional Chinese Medicine (Changzhou Wujin Hospital of Traditional Chinese Medicine), Jiangsu, China

**Keywords:** 16S rRNA, lower respiratory microbiota, nosocomial infection, upper respiratory microbiota, ventilator-associated pneumonia

## Abstract

**Background and objective:**

Ventilator-associated pneumonia (VAP) is a leading cause of morbidity and mortality in critically ill patients receiving mechanical ventilation for over 48 hours. This study aims to explore the spatiotemporal dynamics of respiratory microbiota in the upper (URT) and lower respiratory tracts (LRT) across three phases, including intubated controls, a newly defined VAP pre-occurrence phase, and confirmed VAP to identify early diagnostic biomarkers and enhance understanding of VAP pathophysiology.

**Methods:**

We analyzed 16S rRNA gene sequencing data from 123 samples retrieved from public database. Raw data were processed using the DADA2 pipeline for quality control and denoising. Taxonomy was assigned using the Silva 138 rRNA database. Alpha and beta diversity metrics were calculated, and LEfSe analysis was employed to identify differentially abundant taxa. The diagnostic potential of key bacterial genera was evaluated using ROC curve analysis, with precision, sensitivity, and specificity values reported.

**Results:**

URT alpha diversity increased significantly in VAP groups compared to controls and pre-VAP phase groups, with no significant changes in LRT alpha diversity. Beta diversity analysis revealed distinct microbial community structures across all three disease stages in both URT and LRT. Key genera, including Prevotella, Leptotrichia, and Peptostreptococcus, were identified as potential early diagnostic biomarkers, with high precision, sensitivity, and specificity. Microbial translocation from the URT to the LRT became increasingly synchronized by day 7 post-intubation, suggesting a critical window for intervention.

**Conclusions:**

This study highlights the dynamic changes in respiratory microbiota during VAP progression and identifies potential microbial biomarkers for early detection. These findings pave the way for microbiota-based diagnostic strategies and targeted interventions. Future research should validate these biomarkers in independent cohorts and explore their functional roles in VAP pathogenesis. Monitoring URT-LRT microbial interactions could enable proactive management of this devastating infection.

## 1 Introduction

Ventilator-associated pneumonia (VAP) represents a critical challenge in intensive care settings, affecting up to 20% of critically ill patients subjected to invasive mechanical ventilation and accounting for significant morbidity, mortality, and healthcare costs (Papazian et al., 2020). Despite advances in clinical management, VAP continues to present diagnostic complexities and therapeutic hurdles. The pathogenesis of VAP involves multifactorial interactions between host factors, microbial communities, and environmental influences. Recent evidence has increasingly implicated respiratory microbiota dysbiosis as a key contributor to VAP development (Zakharkina et al., 2017; Lamarche et al., 2018; Emonet et al., 2019).

The intricate relationship between the respiratory microbiota and host immune responses plays a pivotal role in the pathophysiology of VAP. Disruptions in the balance of microbial communities can foster an environment conducive to pathogenic colonization and infection (Kitsios et al., 2017). However, existing research predominantly focuses on static snapshots of microbial composition, with limited exploration of the dynamic spatiotemporal evolution of microbial communities across different anatomical regions of the respiratory tract and how these changes might predate clinical manifestations of VAP.

To address this gap, our study introduces the concept of a “VAP pre-occurrence phase”, bridging the interval between intubation and overt VAP manifestation. By re-grouping a publicly available 16S rRNA dataset into defined temporal stages (control, pre-VAP, and VAP) and performing integrative analysis, we systematically investigated the migration, colonization, and interactions of microbial communities within the upper (URT) and lower respiratory tracts (LRT). This study was specifically designed to define conserved, stage-specific microbial signatures across the VAP development continuum, providing a foundational framework for future high-resolution temporal analyses. This approach not only enhances our understanding of VAP pathophysiology but also explores the potential of these microbial dynamics as early predictive and diagnostic biomarkers, offering avenues for timely interventions to mitigate this devastating nosocomial infection.

## 2 Methods

### 2.1 Dataset acquisition and study inclusion criteria

A systematic searches for public available data were performed on the NCBI Sequence Read Archive (SRA, http://www.ncbi.nlm.nih.gov/sra), European Nucleotide Archive (ENA, http://www.ebi.ac.uk/ena) and Genome Sequence Archive (GSA, https://ngdc.cncb.ac.cn/gsa/) database using the search term “ventilator-associated pneumonia”, “VAP”, “ventilator-associated lower respiratory tract infection”, “VA-LRTI”, “mechanical ventilation”, “endotracheal tube” and “16S rRNA”, and limiting search results by “Bioproject” until February 2025. Bioproject accession numbers containing high throughput sequencing reads and associated metadata were collected. Then, we retrieved articles from NCBI PubMed or Google Scholar using submitted Bioproject information. Included studies had to meet the following criteria: (a) they had to involve samples obtained from the human respiratory tract; (b) the results of the microbiota data were obtained by 16S rRNA gene sequencing; (c) the use of tracheal intubation prior to sampling was documented; and (d) there were relevant metadata, sequencing data, and barcodes available from public database. In order to facilitate comparisons of data across studies, a preformatted metadata file including sample ID, sequencing type, country and diagnosis was collected from public database. Finally, all samples were derived from the same public dataset [PRJEB26875 https://www.ncbi.nlm.nih.gov/bioproject/?term=PRJEB26875 (Sommerstein et al., 2019)]; no additional external database were merged. Patients with acute critical illness and anticipated duration of mechanical ventilation >4 days were eligible. The downloaded datasets were grouped according to the following stages: intubated control, pre-VAP phase (VAP pre-occurrence phase: from the start of 48 hours of invasive mechanical ventilation until 24 hours before the first occurrence of clinical VAP diagnostic criteria) and VAP. For details on the grouping of all samples, please refer to the supplementary information **Supplementary Table S1**. (URT samples: upper respiratory tract samples, specifically referring to oropharyngeal samples; LRT samples: lower respiratory tract samples, specifically referring to tracheobronchial secretion samples).

### 2.2 Sequence processing

The raw data from all datasets were downloaded in the sequence read archive (SRA) and converted into fastq format using SRA Toolkit. Next, all downloaded 16S rRNA gene sequences were processed using the open-source DADA2 package (version 1.26) for quality control and denoising using a parametric error model in R (version 4.3.3). Key DADA2 parameters were set as follows: truncQ = 2, maxEE = c(2,2), truncLen = c(225, 200) based on the quality profile of forward and reverse reads, and minLen = 50. The filtering step removed reads with expected errors >2. Filtering, learning errors, dereplication, amplicon sequence variant (ASV) inference and chimera removal were performed with default settings unless otherwise specified. To standardize sequencing depth across samples, rarefaction was performed at 10,000 reads per sample. Low-abundance ASVs (total count <2 across all samples and present in <10% of samples) were removed prior to downstream analysis. The relevant R analysis code has been uploaded to the GitHub repository and can be accessed at https://github.com/xiaochenxiaochenxinxiangshicheng/16s.

### 2.3 Statistical analysis

Data were analyzed using R4.3.3 software. Sample Shannon and Simpson indices were calculated based on species annotation results to analyze colony α-diversity. Pairwise comparisons were performed using Wilcoxon rank-sum test. Colony β-diversity was assessed by principal component analysis (PCoA) based on the Bray-Curtis distance, and permutational multivariate analysis of variance (PERMANOVA) was performed. Based on the results of species annotation, all the genera were merged at the genus level, and all the colonies that could not be categorized into one genus were classified as unclassified. 15 genera with the highest relative abundance were retained, and the remaining genera were merged and expressed as other, and the stacked histograms were plotted. Raw data underwent relative abundance analysis and subsequent linear discriminant analysis (LDA effect value, LEfSe) to screen for significantly different groups across levels, using an LDA value >3 as the screening criterion. To control for false positives due to multiple comparisons, the false discovery rate (FDR) method was applied, and only taxa with an adjusted P-value (q) < 0.1 were considered statistically significant. Multi-group difference volcano plot analysis was employed to explore the differential distribution of bacterial abundance at the genus level among multiple groups. Differences in colony expression were assessed using the DESeq method. The diagnostic value of the bacterial groups for VAP was analyzed by plotting ROC curves with the true positive rate (sensitivity) as the vertical coordinate and the false positive rate (1-specificity) as the horizontal coordinate. The Hmisc software package v4.7 was loaded and correlations between upper respiratory microbiota abundance with lower respiratory microbiota abundance calculated using Spearman correlation analysis. Statistical analyses were performed using SPSS^®^ Statistics v27, and plotting was performed using the corrplot, ggplot2, and ggpubr packages in R4.3.3.

## 3 Results

### 3.1 Alpha and beta diversity differences

**Figure 1:**
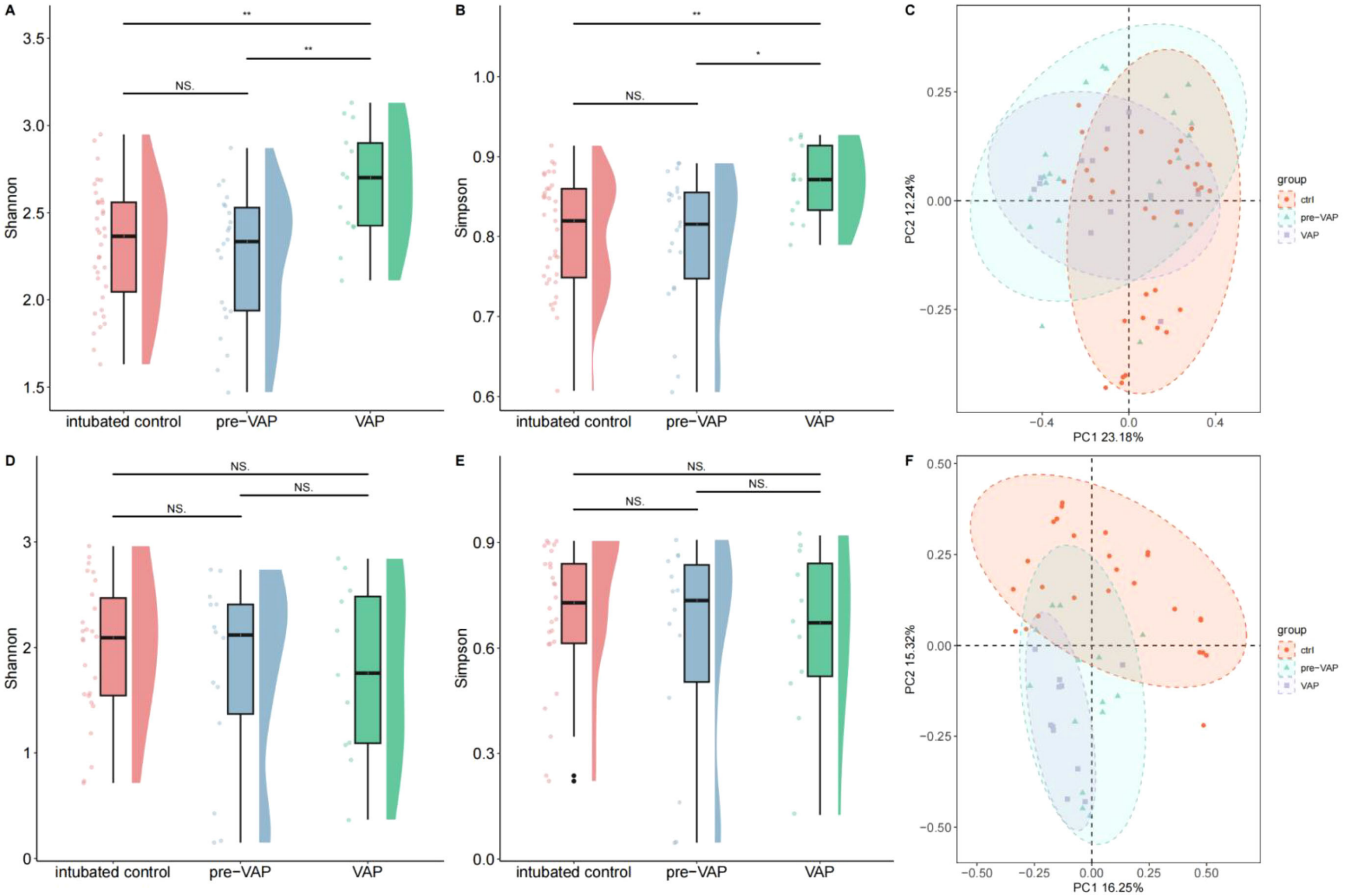
Analysis of microbiota diversity in upper respiratory tract (URT) and lower respiratory tract (LRT) samples. **(A, D)** Alpha-diversity estimated from the Shannon index. **(B, E)** Alpha-diversity estimated from Simpson index. The solid black line indicated the corresponding median value in each group. Pairwise comparisons were performed using Wilcoxon rank-sum test. Asterisks mean differences between the two groups are statistically significant. NS., no significant difference. **(C, F)** Principal coordinate analysis (PCoA) for all included samples based on Bray-Curtis distance. P-value was estimated by permutational multivariate analysis of variance (PERMANOVA). NS, no significant difference; *, P < 0.05; **, P < 0.01.

Our analysis revealed a clear and significant progression in the ecological complexity of the upper respiratory tract (URT) microbiota along the VAP development continuum. Specifically, we observed markedly higher Shannon diversity (*P* < 0.01, **Figure 1A**) and Simpson diversity (*P* < 0.01, **Figure 1B**) in the VAP group compared to both the intubated controls and pre-VAP phase groups. While the difference between control and pre-VAP phase groups did not reach statistical significance, the overall trend of increasing diversity from control to pre-VAP phase to VAP was statistically significant (*P* for trend < 0.05), indicating a progressive disruption of community structure preceding clinical VAP diagnosis. In the lower respiratory microbiota, we found no significant difference in alpha diversity between all groups (*P*>0.05, **Figures 1D, E**). We next analyzed whether there were differences in the structures of microbial communities associated with different disease stages. Beta diversity was visualized by principal coordinate analysis (PCoA) based on the Bray-Curtis distance. Significant differences in the microbial community structure of the upper respiratory tracts (PERMANOVA, F = 4.06, *P <* 0.001, **Figure 1C**) and lower respiratory tracts (PERMANOVA, F = 2.72, *P <* 0.01, **Figure 1F**) were observed in all three disease stages.

### 3.2 Characteristics of the upper and lower respiratory microbiota

**Figure 2:**
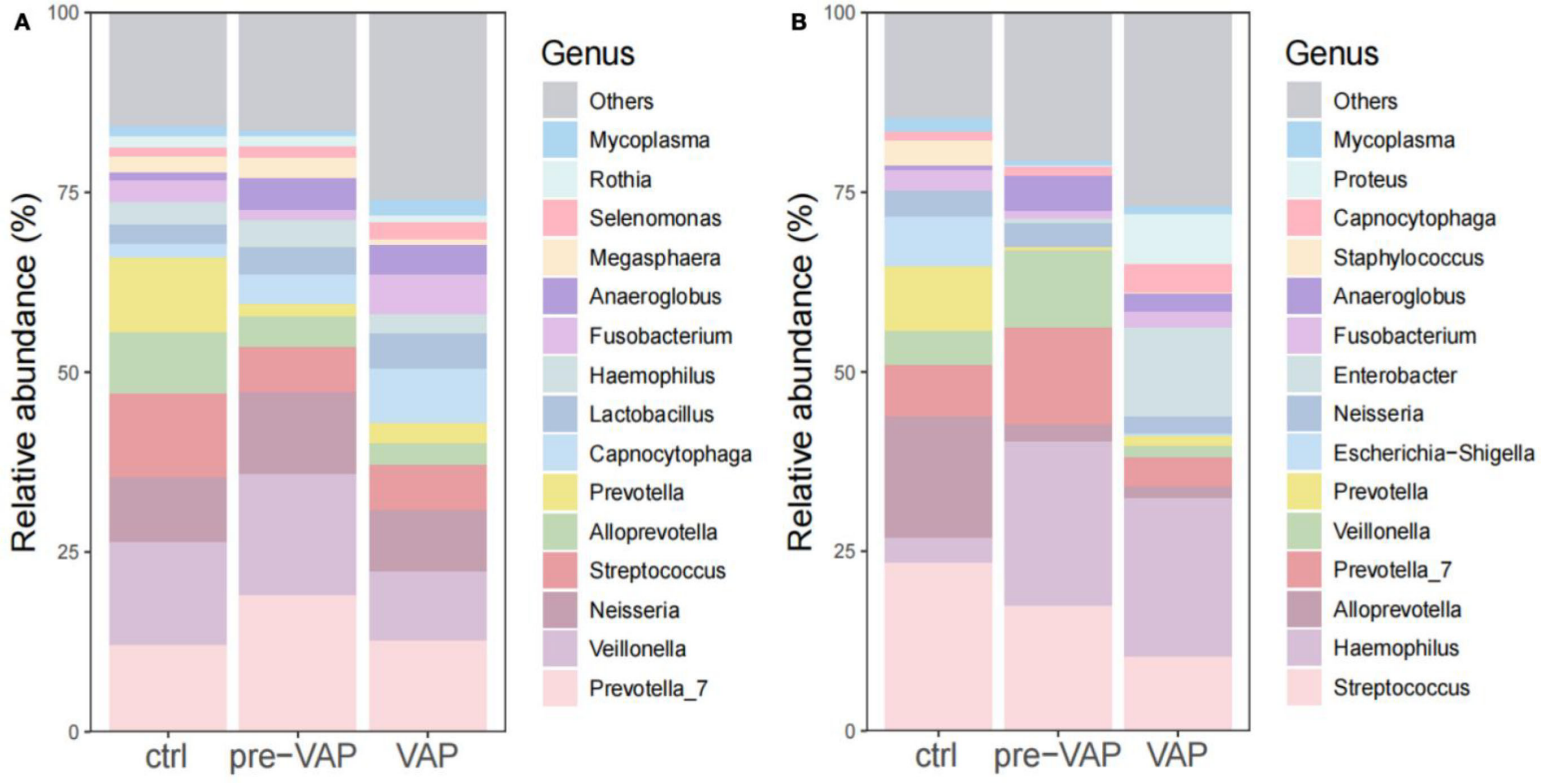
Horizontal structural analysis of respiratory bacteria genera. **(A)** Stacked bars of different groups represent the relative abundance of upper respiratory bacteria genera. **(B)** Stacked bar graphs of different groups representing the relative abundance of lower respiratory tract bacterial genera.

This study investigated the integrated respiratory microbial composition during the course of VAP. At the phylum level, the upper and lower respiratory tract microbiota were mainly composed of the bacterial phyla Firmicutes, Bacteroidota, Proteobacteria, Actinobacteriota and Fusobacteriota. Hierarchical clustering analysis showed that the distribution of the first nine phyla of upper and lower respiratory tract microorganisms was similar (**Supplementary Figure S1**). Among them, Firmicutes was the dominant phylum, which played a key role in maintaining the microecological balance of the respiratory tract. At the genus level, *Prevotella 7*, *Veillonella*, *Neisseria*, *Streptococcus*, *Alloprevotella* and *Prevotella* were the most prevalent and abundant genera of upper respiratory tract micromicrobiota (**Figure 2A**). The lower respiratory tract micromicrobiota is dominated by *Streptococcus*, *Haemophilus*, *Alloprevotella*, *Prevotella 7*, *Veillonella* and *Prevotella* are the most prevalent and abundant genera of lower respiratory tract microbes (**Figure 2B**). There were significantly more genera present in the VAP group, both in the upper and lower respiratory tracts.

### 3.3 Differential taxa across different disease stages in upper respiratory tract

**Figure 3:**
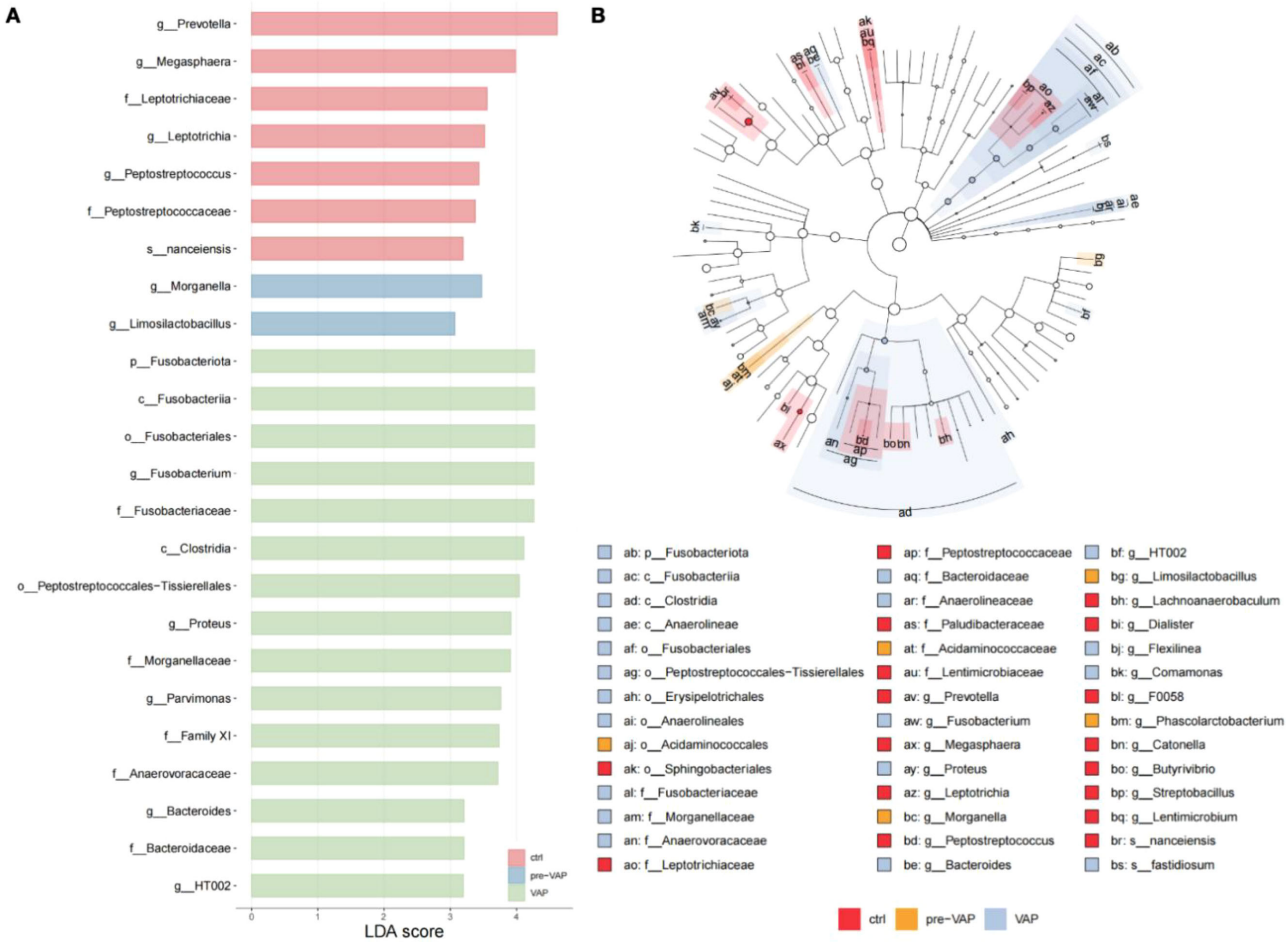
Enriched taxa of the upper respiratory microbiota of the three groups based on LEfSe analysis. **(A)** Linear discriminant analysis: taxa that are least enriched between the sexes according to the LDA score (log 10) (effect size estimate) are indicated in the bar graph. Only taxa meeting P < 0.05 and the LDA score significance threshold |> 3| are shown. Colors represent groups where the taxa shown are more enriched relative to the remaining two groups. **(B)** Branching diagram: phylum, order, family and genus level from center to outside. In the taxonomic branching diagram, each successive circle represents a different phylogenetic level.

Through LEfSe analysis, a total of 24 microbial taxa with significant differences among groups and LDA scores greater than 3 were identified in the upper respiratory tract (oropharyngeal) samples (adjusted *P* < 0.05, FDR q < 0.1) (**Figure 3**). Among these, 7 taxa were enriched in the control group, 2 in the pre-VAP phase group, and 15 in the VAP group. At the phylum level, the control group was characterized by enrichment of Bacteroidota and Fusobacteriota (LDA = 4.63 and 4.35, respectively), whereas no phylum-level enrichment was observed in the pre-VAP phase group, and the VAP group was significantly enriched for Firmicutes (LDA = 4.12). At the genus level, the control group showed significantly higher relative abundances of *Prevotella* (LDA = 4.63), *Megasphaera* (LDA = 4.06), *Leptotrichia* (LDA = 3.57), and *Peptostreptococcus* (LDA = 3.44), which are common commensals of the oral cavity. The pre-VAP phase group exhibited enrichment of *Morganella* (LDA = 3.47) and *Limosilactobacillus* (LDA = 3.04), potentially representing early transitional signals of dysbiosis prior to VAP onset. The VAP group was enriched for several potentially pathogenic genera, including *Fusobacterium* (LDA = 4.34), *Parvimonas* (LDA = 3.79), *Bacteroides* (LDA = 3.20), and *HT002* (LDA = 3.15), along with *Proteus, Flexilinea, Comamonas, and Desulfovibrio*, indicating a pronounced shift toward a pathogenic and anaerobic-associated microbial community. At the species level, *Prevotella nanceiensis* (LDA = 3.21) was enriched in the control group. The cladogram further illustrated the hierarchical relationships among taxa: the control group markers were mainly distributed within Bacteroidota (e.g., *Prevotella*) and Fusobacteriota (e.g., *Leptotrichia*); the pre-VAP phase group markers were scattered within Proteobacteria (e.g., *Morganella*) and Firmicutes (e.g., *Limosilactobacillus*); and the VAP group markers were concentrated in Firmicutes (class Clostridia, order Peptostreptococcales-Tissierellales) and the genus *Fusobacterium*. Higher LDA scores indicate greater discriminatory power of the corresponding taxon for its respective group. Collectively, these results reveal a dynamic succession of the upper respiratory microbiota from a commensal-dominant profile (control) through a transitional profile (pre-VAP phase) to a pathogen-enriched profile (VAP), supporting the potential use of upper respiratory microbial signatures as early warning indicators of impending VAP.

### 3.4 Differential taxa across different disease stages in lower respiratory tract

**Figure 4:**
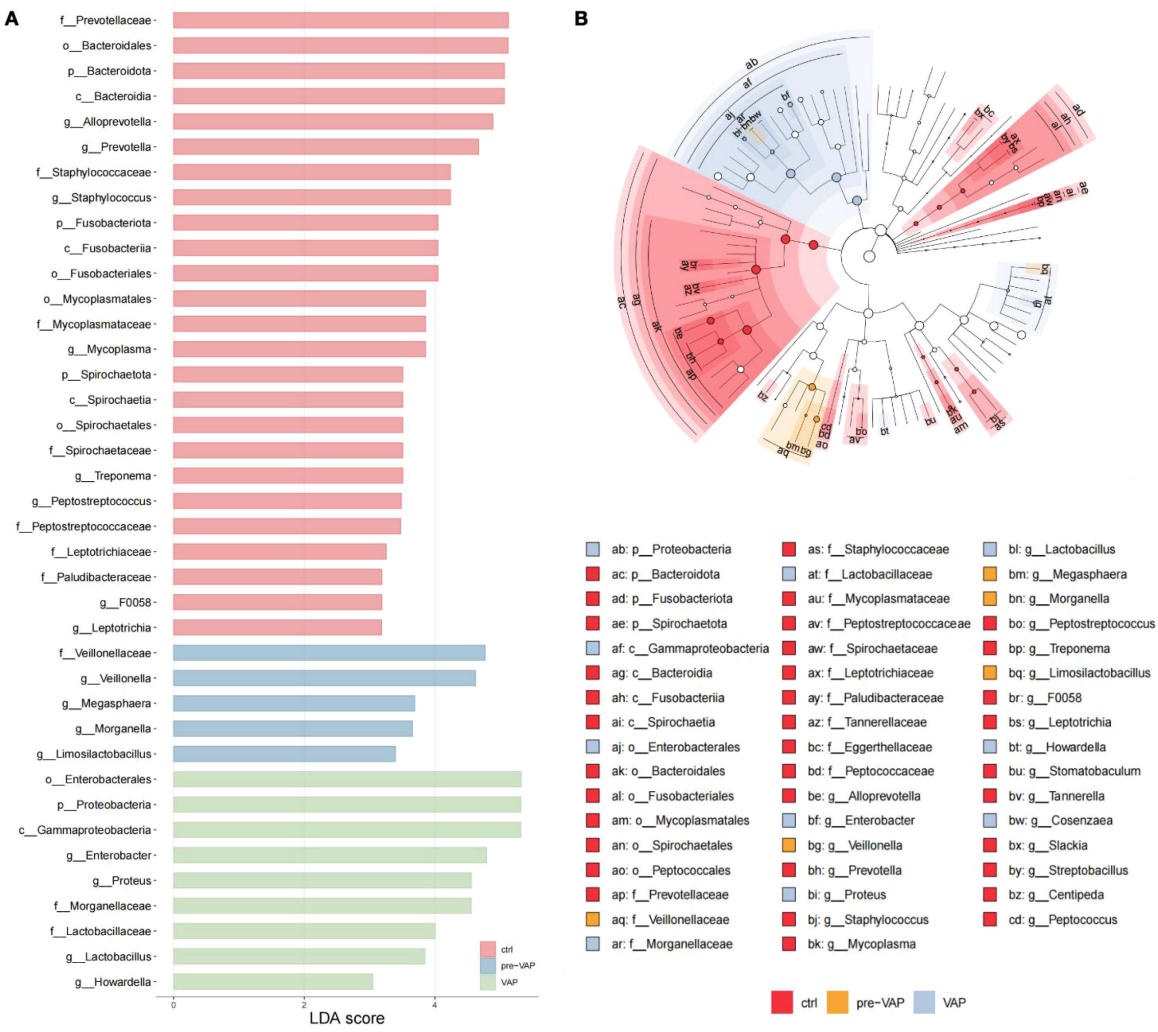
Enriched taxa of the lower respiratory microbiota of the three groups based on LEfSe analysis. **(A)** Linear discriminant analysis: taxa that are least enriched between the sexes according to the LDA score (log 10) (effect size estimate) are indicated in the bar graph. Only taxa meeting P < 0.05 and the LDA score significance threshold |> 3| are shown. Colors represent groups where the taxa shown are more enriched relative to the remaining two groups. **(B)** Branching diagram: phylum, order, family and genus level from center to outside. In the taxonomic branching diagram, each successive circle represents a different phylogenetic level.

Through LEfSe analysis, multiple microbial taxa with significant differences among groups were identified in the lower respiratory tract (endotracheal) samples (adjusted P < 0.05, FDR q < 0.1) (**Figure 4**). At the phylum level, the control group was characterized by enrichment of Bacteroidota (LDA = 5.06), Fusobacteriota (LDA = 4.05), and Spirochaetota (LDA = 3.51); the pre-VAP phase group showed no phylum-level enrichment; and the VAP group was significantly enriched for Proteobacteria (LDA = 5.31). At the family level, the control group was enriched for Prevotellaceae (LDA = 5.13), Staphylococcaceae (LDA = 4.24), Peptostreptococcaceae (LDA = 3.48), Leptotrichiaceae (LDA = 3.25), Paludibacteraceae (LDA = 3.19), Spirochaetaceae (LDA = 3.51), Tannerellaceae (LDA = 2.82), and Peptococcaceae (LDA = 2.51); the pre-VAP phase group was enriched for Veillonellaceae (LDA = 4.77); and the VAP group was enriched for Morganellaceae (LDA = 4.56) and Lactobacillaceae (LDA = 4.01). At the genus level, the control group showed higher relative abundances of Prevotella (LDA = 4.67), Alloprevotella (LDA = 4.89), Staphylococcus (LDA = 4.23), Peptostreptococcus (LDA = 3.48), Treponema (LDA = 3.51), Leptotrichia (LDA = 3.18), F0058 (LDA = 3.19), Peptococcus (LDA = 2.51), Tannerella (LDA = 2.82), and Slackia (LDA = 2.67); the pre-VAP phase group was enriched for Veillonella (LDA = 4.62), Megasphaera (LDA = 3.69), Morganella (LDA = 3.65), and Limosilactobacillus (LDA = 3.39); and the VAP group was enriched for Enterobacter (LDA = 4.79), Proteus (LDA = 4.56), Lactobacillus (LDA = 3.85), and Howardella (LDA = 3.05). The cladogram further illustrated the hierarchical relationships: control group markers were broadly distributed across Bacteroidota, Fusobacteriota, and Spirochaetota; pre-VAP phase group markers were concentrated in Veillonellaceae and its genera Veillonella and Megasphaera; and VAP group markers were concentrated in Proteobacteria, particularly Enterobacterales, Morganellaceae, and Enterobacter.

### 3.5 Potential biomarkers to classify different disease stages in upper respiratory tract

**Figure 5:**
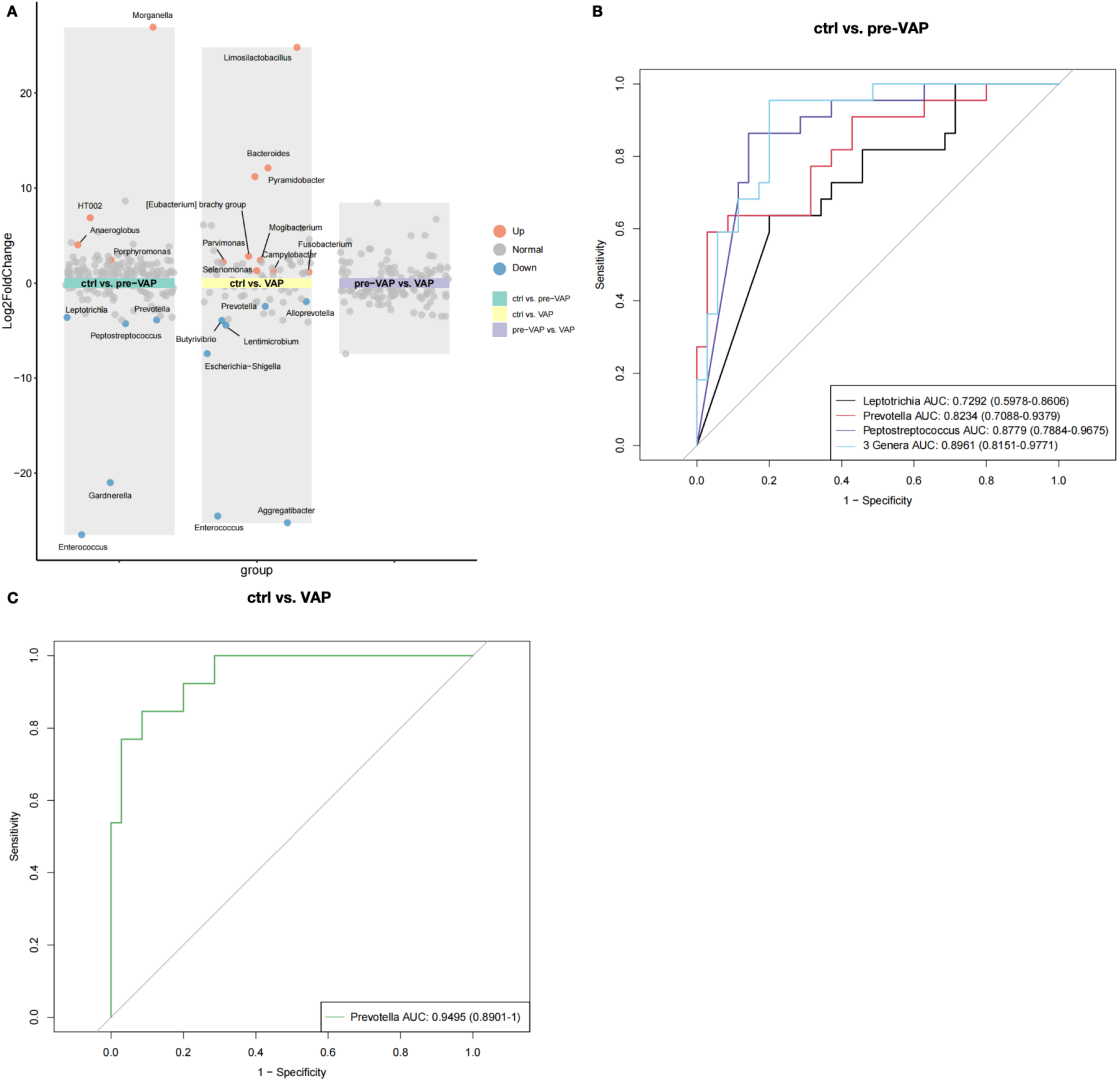
Differential taxa between control, pre-VAP phase, and VAP and the diagnostic genus markers in upper respiratory tract. **(A)** The significantly altered genera as revealed by the DESeq test. **(B, C)** Receiver operating characteristic (ROC) analysis for the identified generic markers with logistic regression model discriminating control, pre-VAP phase and VAP.

A differential analysis of the bacterial genera revealed a significant variation between the two phases of control and pre-VAP phase. Specifically, the pre-VAP phase phase exhibited an enrichment of four genera (*Morganella*, *HT002*, *Anaeroglobus* and *Porphyromonas*) compared to the control phase. Conversely, five genera (*Leptotrichia*, *Prevotella*, *Peptostreptococcus*, *Gardnerella* and *Enterococcus*) were depleted in the pre-VAP phase phase (**Figure 5A**). The potential of bacterial genera with an area under the curve (AUC) > 0.7 as diagnostic biomarkers to distinguish control from VAP was determined by ROC analysis (**Supplementary Figure S2A**). A backward stepwise selection algorithm identified *Leptotrichia*, *Prevotella* and *Peptostreptococcus* as potential biomarkers, and a logistic regression model was developed based on these three biomarkers. The performance of the model was then evaluated using ROC analysis, which yielded an area under the curve (AUC) of 0.8961 (95% CI: 0.8151-0.9771) (**Figure 5B**).

A differential analysis of the genera revealed significant disparities between the control and VAP phases. *Limosilactobacillus, [Eubacterium] brachy group, Mogibacterium, Parvimonas, Bacteroides, Pyramidobacter, Fusobacterium, Selenomonas, and Campylobacter* were enriched in the VAP stage. Conversely, seven genera, namely *Alloprevotella*, *Prevotella*, *Butyrivibrio*, *Lentimicrobium*, *Escherichia-Shigella*, *Aggregatibacter* and *Enterococcus* exhibited a decrease in abundance (**Figure 5A**). Utilizing a receiver operating characteristic (ROC) analysis, we ascertained the diagnostic potential of bacterial genera with an area under the curve (AUC) > 0.7 as biomarkers to differentiate between the control and VAP phases (**Supplementary Figure S2B**). The analysis identified *Prevotella* as a promising diagnostic biomarker, with an area under the curve (AUC) of 0.9495 (95% CI: 0.8901-1) (**Figure 5C**). Furthermore, the study revealed no statistically significant difference in bacilli between the pre-VAP phase and VAP stages (**Figure 5A**).

### 3.6 Potential biomarkers to classify different disease stages in lower respiratory tract

**Figure 6:**
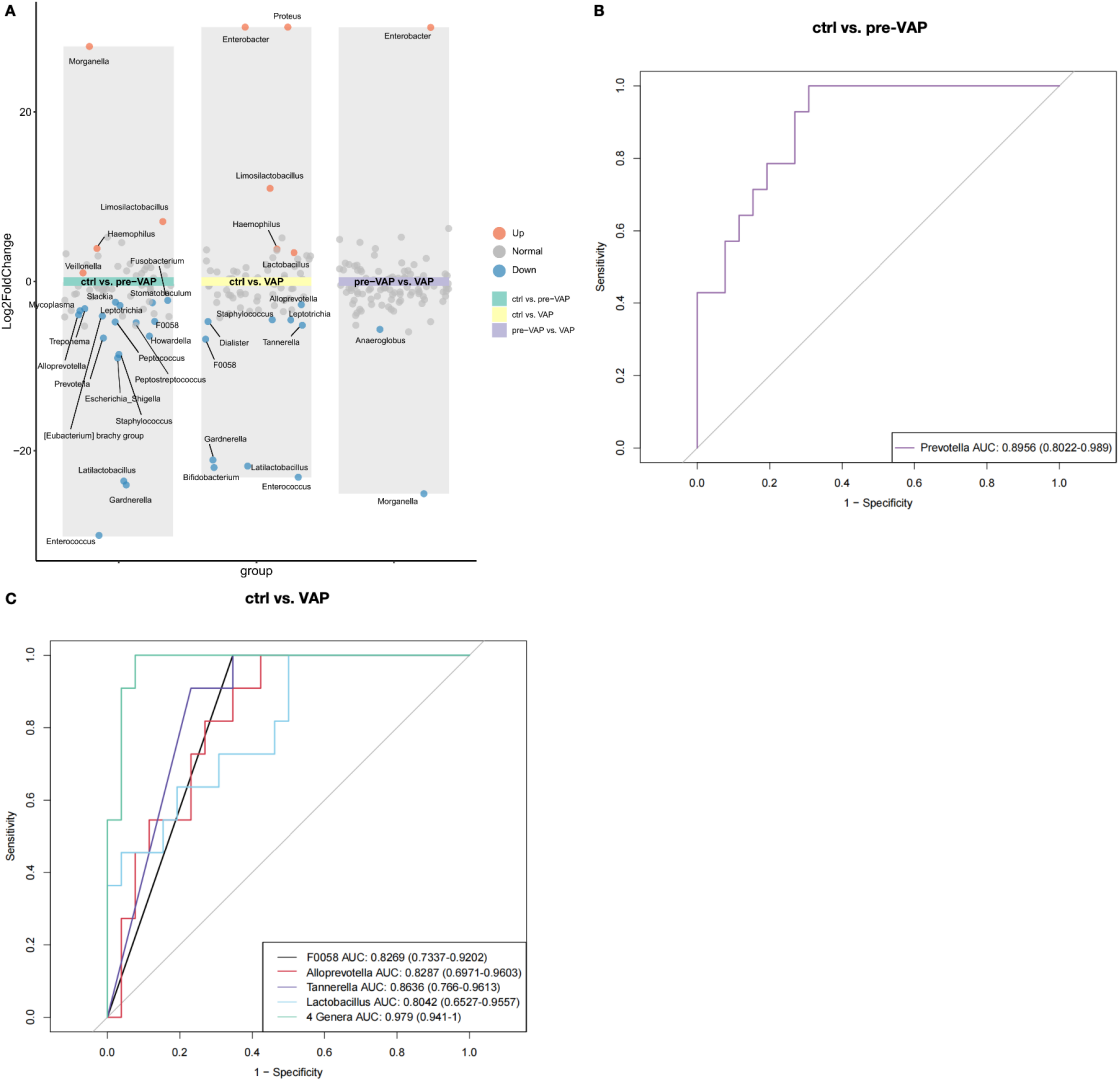
Differential taxa between control, pre-VAP phase, and VAP and the diagnostic genus markers in lower respiratory tract. **(A)** The significantly altered genera as revealed by the DESeq test. **(B, C)** Receiver operating characteristic (ROC) analysis for the identified generic markers with logistic regression model discriminating control, pre-VAP phase and VAP.

Among the genera that differed significantly between the control and pre-VAP phase phases, four genera, *Morganella*, *Limosilactobacillus*, *Haemophilus* and *Veillonella*, were found to be abundant in the VAP phase compared to the control phase. On the other hand, *Slackia*, *Fusobacterium*, *Stomatobaculum*, *Leptotrichia*, *Treponema*, *Mycoplasma*, *Alloprevotella*, *[Eubacterium] brachy group*, *F0058*, *Peptococcus*, *Peptostreptococcus*, *Howardella*, *Prevotella*, *Staphylococcus*, *Escherichia-Shigella*, *Latilactobacillus*, *Gardnerella* and *Enterococcus* were depleted (**Figure 6A**). Using ROC analysis, we determined the potential of bacterial genera with AUC > 0.7 as diagnostic biomarkers to discriminate control from VAP stage (**Supplementary Figure S2C**). *Prevotella* was identified as a potential biomarker using a backward stepwise selection algorithm, yielding an area under the curve (AUC) of 0.8956 (95% CI: 0.8022-0.989) (**Figure 6B**).

Among the genera with significant differences between the control and VAP phases, five genera *Enterobacter*, *Proteus*, *Limosilactobacillus*, *Haemophilus* and *Lactobacillus* were found to be enriched in the pre-VAP phase phase compared to the control phase. On the other hand, *Alloprevotella*, *Staphylococcus*, *Leptotrichia*, *Dialister*, *Tannerella*, *F0058*, *Gardnerella*, *Latilactobacillus*, *Bifidobacterium* and *Enterococcus* were depleted (**Figure 6A**). ROC analysis was used to determine the potential of genera with AUC > 0.7 as diagnostic biomarkers to discriminate control from VAP (**Supplementary Figure S2D**). *F0058*, *Alloprevotella*, *Tannerella* and *Lactobacillus* were identified as potential biomarkers using a backward stepwise selection algorithm, and logistic regression models were constructed based on these four biomarkers. To evaluate the performance of the model, ROC analysis was performed, which yielded an area under the curve (AUC) of 0.979 (95% CI: 0.941-1) (**Figure 6C**). Significantly different genera existed between pre-VAP phase and VAP stages (**Figure 6A**), but no genera with AUC > 0.7 were found by ROC analysis (**Supplementary Figure S2E**).

### 3.7 Spatial differences and associations in the respiratory microbiota

**Figure 7:**
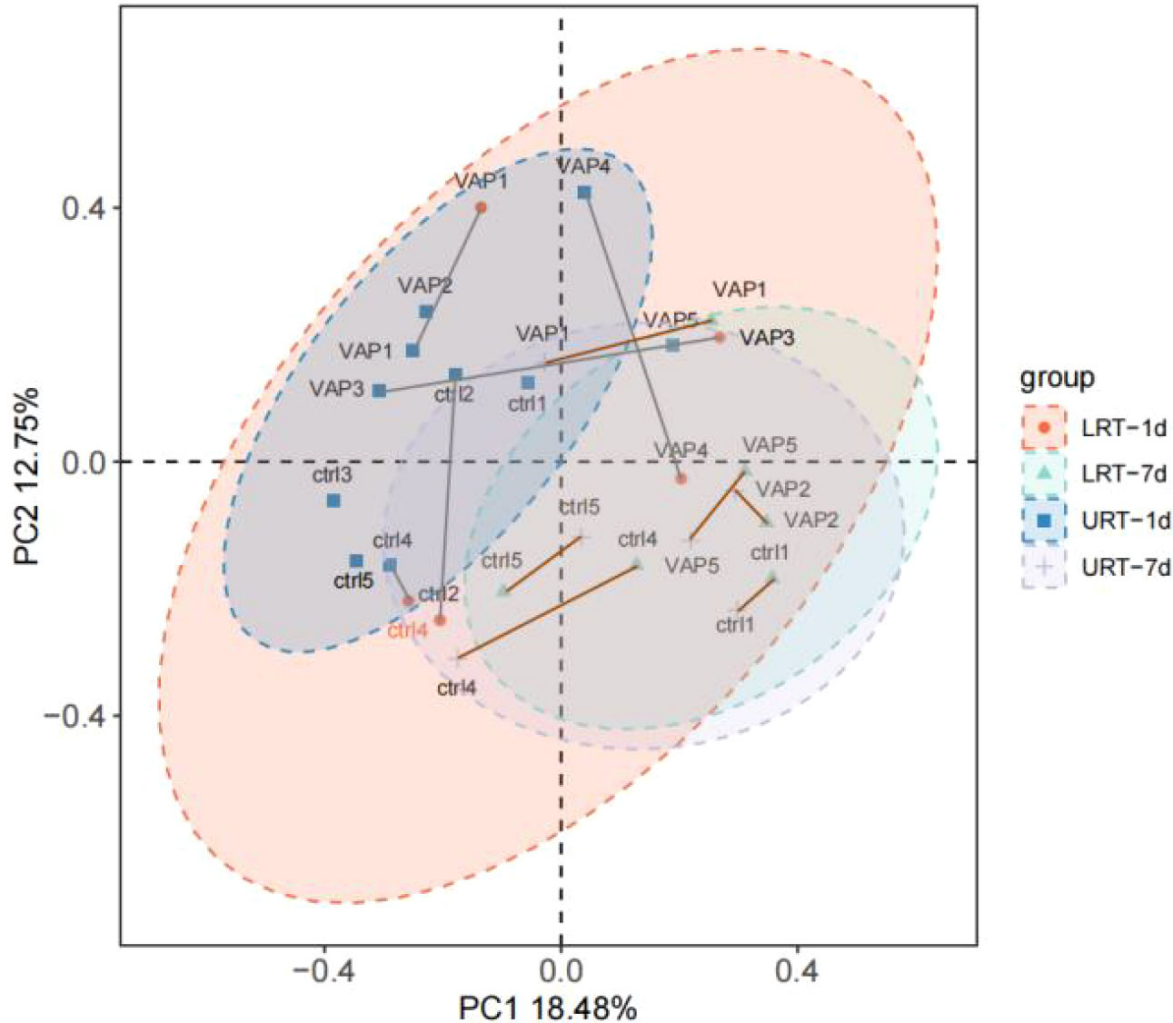
Associations between microbes in different niches of the respiratory tract. The connected points represent paired URT and LRT samples collected from the same patient at the same time point. The length of each connecting line visually represents the Bray-Curtis dissimilarity between the upper and lower respiratory tract niches for that specific patient-timepoint combination.

After describing the different microbial compositions of the various stages of the respiratory tract, we estimated the spatial dynamics of the respiratory microbiota of the same patient by selecting samples from the first day of sampling and the seventh day of sampling. Regarding the beta-diversity based on the Bray-Curtis distance, the microbiota in the upper and lower respiratory tracts on the first and seventh day showed different clustering (PERMANOVA, F = 2.3801, *P <* 0.001), and the similarity of the microorganisms in the upper and lower respiratory tracts on the seventh day was significantly higher when compared to the similarity of the upper and lower respiratory tract microbiota on the first day (**Figure 7**).

**Figure 8:**
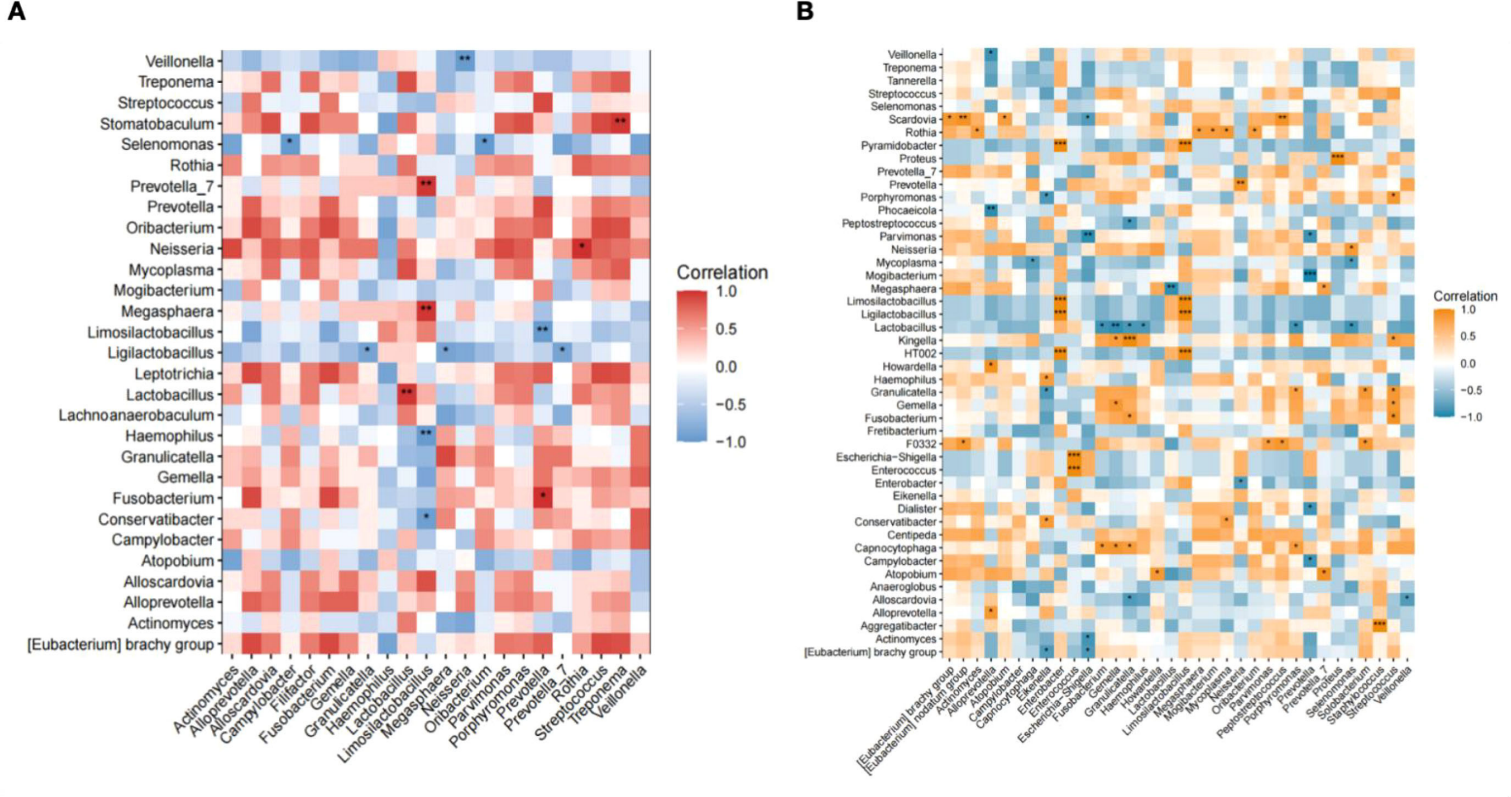
Heat map of genus-level interactions between upper and lower respiratory tract microorganisms. **(A)** Spearman correlation between upper respiratory tract bacterial genera (y-axis) and lower respiratory tract bacterial genera (x-axis) in patients on day 1. **(B)** Spearman correlation between upper respiratory tract bacterial genera (y-axis) and lower respiratory tract bacterial genera (x-axis) in patients on day 7. * indicates P < 0.05, ** indicates P < 0.01, *** indicates P < 0.001.

In the samples taken on the first day of sampling, we found the existence of correlations between bacterial genera of the upper respiratory tract and those of the lower respiratory tract. For example, Prevotella_7, *Megasphaera* in the upper respiratory tract with *Limosilactobacillus* in the lower respiratory tract; *Stomatobaculum* with *Treponema*; *Neisseria* with *Rothia*; *Lactobacillus* with Lactobacillus; *Fusobacterium* with *Prevotella*; were a positive correlation (*P <* 0.05). *Haemophilus*, *Conservatibacter* in the upper respiratory tract with *Limosilactobacillus* in the lower respiratory tract; *Selenomonas* with *Campylobacter*, *Oribacterium*; *Ligilactobacillus* with *Granulicatella*, *Megasphaera*, Prevotella_7; *Veillonella* with *Neisseria*; *Limosilactobacillus* with *Prevotella*; were negatively correlated (*P <* 0.05, **Figure 8A**).

We found more correlations between upper and lower respiratory genera in samples taken on the seventh day of sampling. For example, *Rothia* in the upper respiratory tract correlated with *Actinomyces*, *Megasphaera*, *Mogibacterium*, *Mycoplasma*, and *Oribacterium* in the lower respiratory tract; *Scardovia* correlated with *[Eubacterium] brachy group*, *[Eubacterium] nodatum group*, *Atopobium*, *Peptostreptococcus*; *F0332* with *[Eubacterium] nodatum group*, *Parvimonas*, *Peptostreptococcus*, *Solobacterium*; *Capnocytophaga* with *Fusobacterium*, *Gemella*, *Granulicatella*, *Porphyromonas*; *Kingella* with *Gemella*, *Granulicatella*, *Streptococcus*; *Pyramidobacter* with *Enterobacter*, *Limosilactobacillus*; *Granulicatella* with *Porphyromonas*, *Solobacterium*; *Conservatibacter* with *Eikenella*, *Mycoplasma*; *Atopobium* with *Howardella*, *Prevotella_7*; *Limosilactobacillus*, *Ligilactobacillus*, *HT002* with *Enterobacter*, *Limosilactobacillus*; *Granulicatella*, *Gemella*. *Fusobacterium* with *Streptococcus*; *Howardella*, *Alloprevotella* with *Alloprevotella*; *Haemophilus*, *Conservatibacter* with *Eikenella*; *Escherichia -Shigella*, *Enterococcus* with *Enterococcus*; *Alloprevotella* with *Alloprevotella*; *Proteus* with *Proteus*; *Prevotella* with *Neisseria*; *Porphyromonas* with *Streptococcus*; *Neisseria* with *Selenomonas*; *Megasphaera* with *Prevotella_7*; *Gemella* with *Gemella*; *Fusobacterium* with *Granulicatella* showed positive correlation (*P <* 0.05). *Parvimonas*, *Mogibacterium*, *Dialister*, *Campylobacter* in upper respiratory tract correlated with *Prevotella* in lower respiratory tract; *Lactobacillus* with *Fusobacterium*, *Gemella*, *Granulicatella*, *Haemophilus*, *Porphyromonas*, *Selenomonas*; *Scardovia*, *Parvimonas*, *Actinomyces*, *[Eubacterium] brachy group* with *Escherichia-Shigella*; *Porphyromonas*, *Granulicatella*, *[Eubacterium] brachy group* with *Eikenella*; *Veillonella*, *Phocaeicola* with *Alloprevotella*; *Peptostreptococcus*, *Alloscardovia* with *Granulicatella*; *Mycoplasma* with *Capnocytophaga*, *Selenomonas*; *Alloscardovia* with *Veillonella*; *Megasphaera* with *Lactobacillus*; *Enterobacter* with *Neisseria* showed negative correlation (**Figure 8B**, *P* < 0.05).

## 4 Discussion

VAP is a multifactorial disease involving interactions between host, microbial, and environmental factors. It occurs in 5%–40% of patients receiving mechanical ventilation for over 2 days and represents a common and serious complication in intensive care units, affecting 8–28% of mechanically ventilated patients, with significant associations with morbidity, mortality, and increased healthcare costs (American Thoracic Society and Infectious Diseases Society of America, 2005; Reignier et al., 2013). The pathogenesis of VAP is closely linked to the dynamic balance of respiratory tract microbial communities, particularly the microbiota “shift” between the upper and lower respiratory tracts, and disruption of this balance leading to lower respiratory tract dysbiosis is recognized as a key risk factor for VAP (Natalini et al., 2023) (Bustos et al., 2023). Existing studies have mainly focused on static snapshots of microbial composition, with limited investigation into the spatiotemporal dynamic evolution of microbial communities and their potential as early predictive biomarkers for VAP; moreover, the precise temporal and spatial changes of these communities preceding VAP onset remain incompletely understood, prompting our study to systematically explore microbial dynamics across distinct stages of VAP development.

Analysis of public datasets revealed significant differences in upper respiratory tract (URT) microbiota diversity and richness among intubated controls, pre-VAP phase, and overt VAP groups, consistent with previous reports that URT microbiota diversity increases with disease progression (Emonet et al., 2019). The URT, including the nasopharynx and oropharynx, exhibits high bacterial colonization with topographical variation in microbial composition (Natalini et al., 2023; Li et al., 2025; Tan et al., 2025); for example, the oropharyngeal microbiota of intubated controls is dominated by Prevotella 7, Veillonella, Neisseria, Streptococcus, Alloprevotella, and Prevotella, some of which such as Veillonella and Streptococcus can induce the production of IL-6, IL-8, IL-10, and TNF-α, indicating that the URT is not merely a passive colonization site but actively contributes to VAP pathogenesis by establishing early dysbiosis that predisposes to subsequent lung invasion (de Steenhuijsen Piters et al., 2020) (van den Bogert et al., 2014; Frey et al., 2021). LEfSe analysis identified Prevotella as enriched in controls, while Leptotrichia, Prevotella, and Peptostreptococcus served as key biomarkers distinguishing intubated controls from the pre-VAP phase phase (AUC > 0.8), and Prevotella also differentiated controls from VAP cases (AUC = 0.9495).

In contrast to the dynamic URT microbiota, the lower respiratory tract (LRT) displayed relatively stable diversity across groups despite significant alterations in composition, with lower biomass but comparable dominant taxa including Streptococcus, Haemophilus, Alloprevotella, Prevotella 7, Veillonella, and Prevotella (Natalini et al., 2023). LEfSe showed Prevotella enrichment in controls, whereas F0058, Alloprevotella, Tannerella, and Lactobacillus were diagnostic biomarkers for VAP. Evidence suggests VAP development is more related to the disruption of specific mutualistic microbes than overall biodiversity loss (Bustos et al., 2023; Filipiak et al., 2024; Guo et al., 2025).

Comparative LEfSe analysis between the oropharynx and trachea uncovered distinct spatial patterns reflecting anatomical and physiological niche differences. The control group presented 25 enriched taxa in the trachea versus only 7 in the oropharynx, suggesting a more heterogeneous baseline microbial community in the LRT of intubated patients. The VAP group showed marked enrichment of Gammaproteobacteria (including Enterobacter and Proteus) exclusively in the trachea, aligning with the clinical definition of VAP as a primarily LRT infection and indicating that Gram-negative pathogen overgrowth in the trachea marks a critical transition point. The pre-VAP phase phase exhibited only two enriched genera (Morganella and Limosilactobacillus) in the oropharynx but three (Veillonella, Megasphaera, and Limosilactobacillus) in the trachea, implying that early microbial signals of impending VAP arise earlier in the trachea and supporting the value of direct tracheal sampling for improved early prediction. These spatial differences emphasize that oropharyngeal and tracheal samples should be analyzed separately rather than pooled as a single respiratory niche.

### 4.1 Contradictions and critical analysis

While our findings corroborate previous observations on the role of Prevotella in respiratory health, there are some contradictions and areas needing further clarification (Könönen and Gursoy, 2021). Some studies suggest that higher Prevotella levels in the URT are associated with health rather than disease states like pneumonia or asthma (Wu et al., 2021; Horn et al., 2022). However, our study identified Prevotella as a diagnostic marker distinguishing control groups from VAP cases, indicating its potential dual role in respiratory health and disease. This duality might be context-dependent, influenced by factors like microbial community structure, host immune status, and environmental exposures.

Moreover, the mechanisms by which Prevotella and other identified biomarkers interact with the host and respiratory pathogens remain to be fully elucidated (Larsen et al., 2012; Bertelsen et al., 2021; Könönen et al., 2022). Although some studies indicate that Prevotella species can enhance protection against respiratory pathogens through immune activation, the exact pathways and their relevance in VAP pathogenesis require deeper investigation (Segal et al., 2013; Segal et al., 2016).

### 4.2 Limitations and future directions

Our study has several limitations. First, the sample of 10 patients, while generating 123 high-density longitudinal and paired samples, restricts generalizability and statistical power for detecting weak microbial associations. Our deep-phenotyping, hypothesis-generating design prioritized within-individual sampling depth to mitigate interpersonal variation common in microbiome research, necessitating validation in larger prospective cohorts. Second, grouping by disease stage rather than fixed post-intubation timepoints prevented precise delineation of daily microbial ecological succession. Third, integration of public 16S rRNA datasets introduced variability from heterogeneous collection, processing, and sequencing protocols, and taxonomic profiling alone is insufficient compared with functional microbiota characterization. Additionally, our study focused on microbial dynamics without detailed assessment of host immune responses, genetic predisposition, or clinical parameters, whose interactions with microbial shifts are critical to VAP and require further exploration. The absence of external independent cohort validation also limits generalizability.

Notably, paired URT-LRT analysis demonstrated a significant increase in microbial community similarity by day 7 post-intubation compared with baseline. This progressive synchronization between upper and lower respiratory ecosystems indicates a critical window during which the LRT is increasingly colonized by URT taxa, creating a microenvironment conducive to VAP development. Although our study focused on stage-specific microbial signatures, this finding provides important preliminary evidence for the temporal dynamics of respiratory microbiota in mechanically ventilated patients.

Future research should validate these findings in standardized prospective studies powered by effect sizes from this cohort, integrate metagenomic and metatranscriptomic analyses for functional insights, and explore microbe–host immune crosstalk. Daily multi-site sampling starting at intubation is recommended to enable advanced time-series analyses such as microbial trajectory modeling for precise identification of ecological events leading to VAP. The biomarkers and stage definitions identified herein provide a robust hypothesis framework and effect size estimates to justify and focus future high-intensity temporal studies of VAP pathogenesis.

### 4.3 Comparison with the original study by Sommerstein et al. (2019)

We acknowledge the foundational work by Sommerstein et al. (2019). It is essential to clarify that the fundamental distinction between our work and the original study lies in the divergent research entry points. The original study operated through a longitudinal observational lens confined to a single anatomical niche, the oropharynx, to describe the natural temporal evolution of its microbiome. In contrast, our study is anchored in a spatial perspective. We implemented a mandatory anatomical stratification that treats the upper respiratory tract (URT, oropharynx) and lower respiratory tract (LRT, trachea) as two distinct yet interacting ecosystems. To facilitate stage-specific comparisons within this spatial framework, we introduced a disease-stage-based grouping, including a newly defined “pre-VAP phase” ($48$ h post-intubation to $24$ h before clinical VAP). Thus, the pre-VAP phase is not merely a temporal claim but a functional analytical window that allows us to interrogate how URT and LRT microbial communities diverge, migrate, and ultimately synchronize preceding overt pneumonia. Consequently, our work addresses two critical questions unaddressed by the original analysis: (1) the existence of distinct, stage-specific microbial signatures across the URT and LRT, and (2) the intensification of spatial coupling between these niches prior to VAP. The observed taxonomic shifts, such as the identification of Prevotella, Leptotrichia, and Peptostreptococcus as pre-VAP biomarkers, stem directly from this spatially resolved, stage-stratified design rather than a contradictory interpretation of the same dataset. We therefore view our work as a complementary extension that adds a critically needed spatial dimension to the original longitudinal portrait, together advancing a more comprehensive, spatiotemporally resolved model of VAP pathogenesis.

## 5 Conclusions

Our study advances the understanding of VAP pathophysiology by delineating the spatiotemporal dynamics of respiratory microbiota and identifying potential early diagnostic biomarkers. These findings underscore the importance of monitoring microbial communities in mechanically ventilated patients and pave the way for microbiota-based strategies to improve VAP detection and management. Future research should further explore the functional roles of these microbial communities and their interactions with the host immune system to develop targeted therapeutic interventions. The stage-specific microbial signatures identified here provide a foundational framework for developing precisely timed interventions and guiding future high-resolution temporal studies of VAP pathogenesis.

## Data Availability

The original contributions presented in the study are included in the article/**Supplementary Material**. Further inquiries can be directed to the corresponding authors.
