# Supplementary material for: Spatio-temporal differences and associations between upper and lower respiratory microbiota in ventilator-associated pneumonia
Source: Front Cell Infect Microbiol. 2026 Jun 5;16:1757535. doi: 10.3389/fcimb.2026.1757535 (PMC13279321; doi:10.3389/fcimb.2026.1757535)
Supplement: Supplementary file 1 [file Table1.docx]

**Supplementary Information**


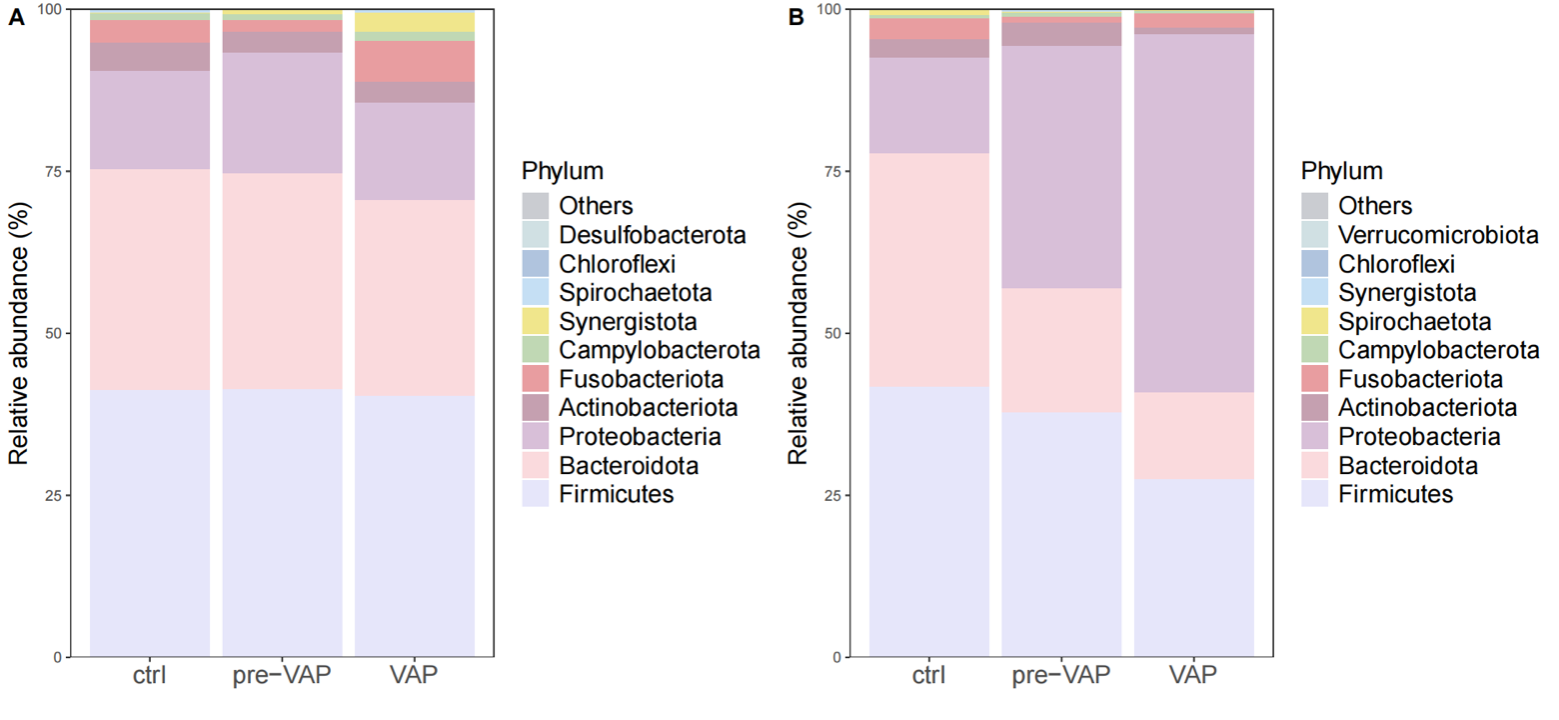


**Figure S1 Horizontal structural analysis of respiratory bacteria phylum.** (A) Stacked bars of different groups represent the relative abundance of upper Respiratory Bacteria phylum. (B) Stacked bar graphs of different groups representing the relative abundance of the lower respiratory tract phylum.


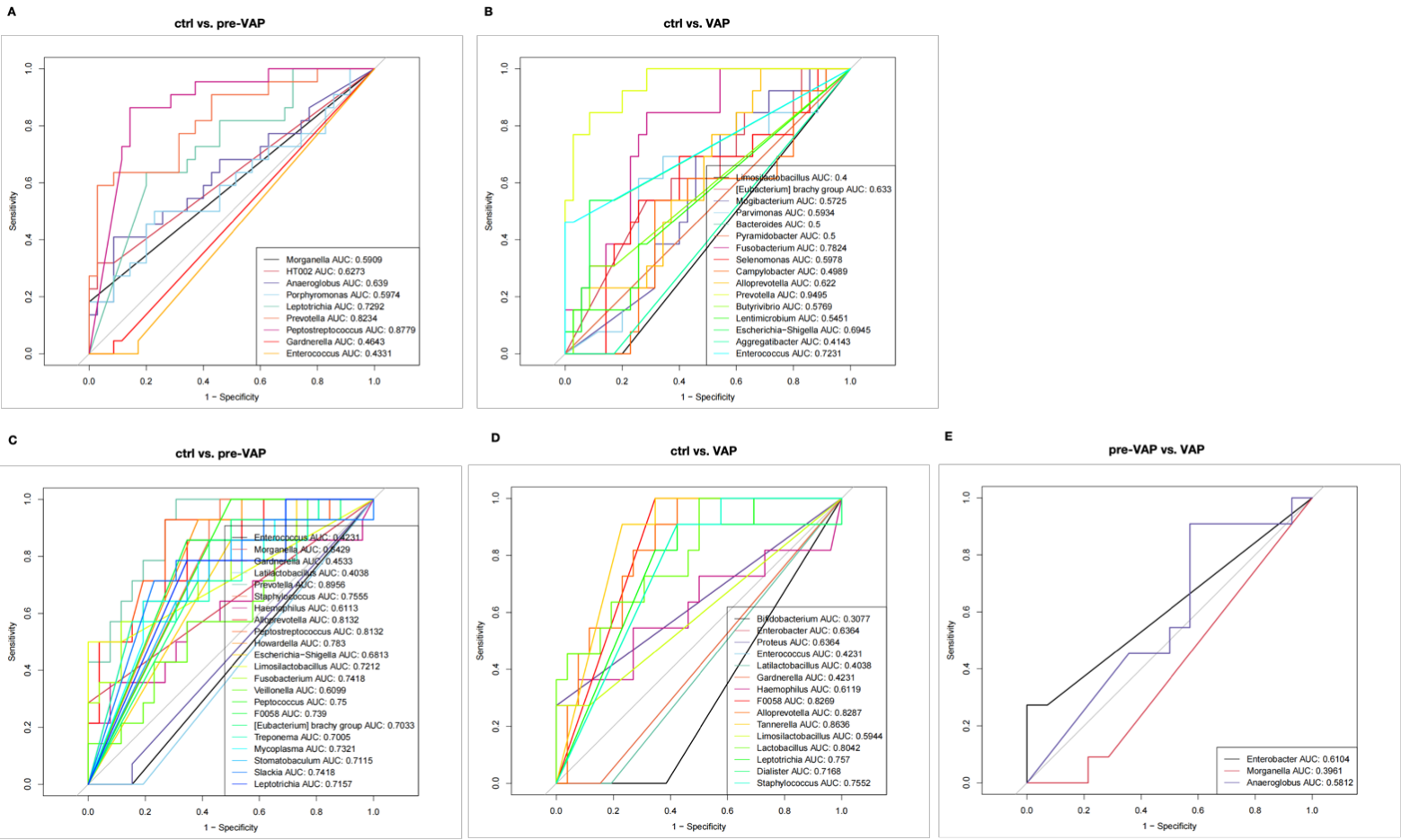


**Figure S2 Receiver operating characteristic (ROC) analysis.** (A-B) Comparison between subgroups of upper respiratory microbiota. (C-E) Comparison between subgroups of lower respiratory microbiota.

| **Table S1：Total sample size of the research subjects** | | | | | |
| --- | --- | --- | --- | --- | --- |
| Sample | Sampling site | NC | pre-VAP | VAP | Total |
| control1 | URT | 9 |  |  | 9 |
| control2 | URT | 5 |  |  | 5 |
| control3 | URT | 5 |  |  | 5 |
| control4 | URT | 7 |  |  | 7 |
| control5 | URT | 9 |  |  | 9 |
| VAP1 | URT |  | 2 | 4 | 6 |
| VAP2 | URT |  | 5 | 4 | 9 |
| VAP3 | URT |  | 6 | 1 | 7 |
| VAP4 | URT |  | 3 | 1 | 4 |
| VAP5 | URT |  | 7 | 2 | 9 |
| control1 | LRT | 7 |  |  | 7 |
| control2 | LRT | 5 |  |  | 5 |
| control3 | LRT | 4 |  |  | 4 |
| control4 | LRT | 4 |  |  | 4 |
| control5 | LRT | 7 |  |  | 7 |
| VAP1 | LRT |  | 2 | 3 | 5 |
| VAP2 | LRT |  | 2 | 3 | 5 |
| VAP3 | LRT |  | 6 | 1 | 7 |
| VAP4 | LRT |  | 3 | 2 | 5 |
| VAP5 | LRT |  | 2 | 2 | 4 |
| Total |  | 62 | 38 | 23 | 123 |
